# Notes on the Treatment of Fœcal Fistula*Abstract from the *Medical Record* of October 24th, 1896.

**Published:** 1897-02

**Authors:** 


### NOTES ON THE TREATMENT OF FÆCAL FISTULÆ*

At the thirteenth annual meeting of the New York State Medical Association, which was recently held in New York City, Dr. Frederick Holme Wiggin, of New York county, presented a paper with the above title. The chief cause of the occurrence of fæcal fistula was stated to be the delay in resorting to operative measures to which patients suffering from typhlenteritis, or strangulated hernia, were frequently subjected while their ailment was carefully diagnosticated. The view recently advanced by a writer on the subject under consideration, that the best treatment for this condition consisted in its prevention, was concurred in. But in the case in which this mishap had occurred, it was pointed out that if the opening was of small size, was located near or below the ileo-cæcal valve and no obstruction to the fæcal current existed, operative measures might be deferred, as in most instances the opening would close in a short time spontaneously. On the other hand, if the bowel opening was of large size, was situated laterally, or some distance above the ileo-cæcal valve, and was accompanied by the escape of a large proportion of the contents of the bowel, operative procedure for the closure of the opening should be speedily undertaken.

The histories of three cases, successfully treated by surgical measures, were cited. In two instances, the patients were inmates of the Hartford (Connecticut) Hospital, and were operated upon by Dr. Wiggin, by reason of an invitation which was extended to him by the medical board of that institution, after several previous unsuccessful efforts to close the bowel openings had been made. The occurrence of the fistulous opening was due in the first case to failure, and in the second case, to delay in resorting to surgical treatment of typhlenteritis, from which disease both patients originally suffered. In the third case, the bowel opening was caused either by the pressure of the gauze used to drain the abscess cavity, or by an ulcerative process which originated from within the gut. In the first case, as the opening in the bowel was of large size, irregular in shape, and the gut was thickened and friable, the diseased portion of bowel containing the opening, about four inches in length, was excised, and the divided ends joined by the suture methods of Maunsell. In

*Abstract from the *Medical Record* of October 24th, 1896.

the second and third cases, the bowel openings were situated in the head of the colon, and were in both instances closed by means of several rows of sutures, after which the omentum was drawn over the former site of the fistula, and retained in position by sutures.

In describing the technic employed, the writer laid much stress upon the following points, viz.: the thorough disinfection of the parts, including the interior of the bowel, with hydrozone, the closing of the intestinal opening, when possible, before the breaking up of the peritoneal adhesions, and the opening of the general cavity, the removal of any existing obstruction to the fæcal current, the disinfection of the bowel surface with a solution of hydrozone, before and after the placing of the sutures, the control of oozing from the cicatricial tissue by the same means and the closure by a single row of silk-worm gut sutures without drainage of the abdominal wound after the washing of the peritoneal cavity with saline solution, some of which is allowed to remain.

In concluding, the writer stated that ever since September, 1893, when he had proved the value of hydrogen dioxide as an effective antiseptic, which in proper solution did not unduly irritate the peritoneum, when followed by a six-tenths per cent. saline solution, he had had little reason to fear the danger of causing septic peritonitis from the accidental escape of pus or fæcal matter while operating; and that when this complication had occurred, it had been invariably successfully met by the use of hydrogen dioxide in the manner described in the paper. He advised the excision of the diseased portion of the gut in those instances where it had become much thickened and friable, and expressed the belief that with a clearer understanding of the objects to be attained by operation—*i. e.* the restoration of the integrity of the intestinal canal, as well as the closure of the opening in the bowel—future operations for the cure of fæcal fistula would more frequently result successfully than they had in the past.

The paper was discussed at some length by Dr. H. O. Marcy, of Boston, and Dr. Joseph D. Bryant, of New York county, who commended it, and in the main, they endorsed the writer's views.

